# Supplementary material for: Timing of increased temperature sensitivity coincides with nervous system development in winter moth embryos
Source: J Exp Biol. 2021 Sep 1;224(17):jeb242554. doi: 10.1242/jeb.242554 (PMC8443866; doi:10.1242/jeb.242554)
Supplement: Supplementary information [file jexbio-224-242554-s1.pdf]

Table S1. Treatment week comparisons of 5°C treatment effect size compared to a constant 10°C for imaging dataset. The difference in effect size between the cold (5°C) and baseline treatment (10°C) was compared between treatment weeks with one-sided hypothesis tests. Estimates in log odds represent the change in the estimated mean difference between treatments (as reported in Table 1) in one week to the next, with the estimated error (Est. Error) and the 95% confidence interval (CI). The evidence ratio (Evid. Ratio) and posterior probability (Post. prob) report the likelihood of a real difference existing (HA) versus no effect (H0). Negative estimates mean a bigger change in the later week compared to the earlier week.

Comparison	Estimate	Est. Error	95% CI			Evid. Ratio	Post. prob*
week 2 – week 3	-0.03	0.53	-0.90	to	0.84	1.10	0.52
week 2 – week 4	-0.04	0.52	-0.87	to	0.80	1.17	0.54
week 2 – week 5	0.77	0.52	-0.08	to	1.61	0.07	0.07
week 2 – week 6	-0.97	0.50	-1.79	to	-0.15	35.07	**0.97**
week 2 – week 7	-1.03	0.51	-1.86	to	-0.19	41.96	**0.98**
week 2 – week 8	-0.78	0.50	-1.59	to	0.04	16.13	0.94
week 2 – week 9	-1.63	0.51	-2.45	to	-0.80	1057.82	**1.00**
week 2 – week 10	-0.88	0.50	-1.68	to	-0.04	22.38	**0.96**
week 2 – week 11	-1.84	0.50	-2.66	to	-1.01	3599.00	**1.00**
week 2 – week 12	-2.12	0.51	-2.96	to	-1.27	Inf	**1.00**
week 2 – week 13	-1.97	0.65	-3.04	to	-0.91	641.86	**1.00**
week 3 – week 4	-0.01	0.52	-0.87	to	0.86	1.04	0.51
week 3 – week 5	0.80	0.52	-0.07	to	1.65	0.07	0.06
week 3 – week 6	-0.94	0.51	-1.77	to	-0.08	28.08	**0.97**
week 3 – week 7	-1.00	0.51	-1.84	to	-0.16	38.56	**0.97**
week 3 – week 8	-0.75	0.51	-1.58	to	0.08	14.00	0.93
week 3 – week 9	-1.60	0.51	-2.44	to	-0.76	749.00	**1.00**
week 3 – week 10	-0.85	0.51	-1.69	to	-0.01	19.88	**0.95**
week 3 – week 11	-1.81	0.51	-2.65	to	-0.97	2570.43	**1.00**
week 3 – week 12	-2.09	0.52	-2.93	to	-1.23	17999.00	**1.00**
week 3 – week 13	-1.94	0.65	-3.01	to	-0.89	691.31	**1.00**
week 4 – week 5	0.81	0.50	-0.02	to	1.62	0.06	0.05
week 4 – week 6	-0.93	0.48	-1.71	to	-0.13	35.51	**0.97**
week 4 – week 7	-0.99	0.49	-1.79	to	-0.20	45.88	**0.98**
week 4 – week 8	-0.74	0.48	-1.52	to	0.04	16.00	0.94
week 4 – week 9	-1.58	0.49	-2.39	to	-0.79	719.00	**1.00**
week 4 – week 10	-0.84	0.49	-1.64	to	-0.04	22.78	**0.96**
week 4 – week 11	-1.80	0.48	-2.59	to	-1.01	3599.00	**1.00**
week 4 – week 12	-2.08	0.49	-2.89	to	-1.28	17999.00	**1.00**
week 4 – week 13	-1.93	0.63	-2.96	to	-0.90	817.18	**1.00**
week 5 – week 6	-1.74	0.49	-2.53	to	-0.92	2570.43	**1.00**
week 5 – week 7	-1.80	0.49	-2.60	to	-0.97	4499.00	**1.00**
week 5 – week 8	-1.55	0.49	-2.33	to	-0.74	719.00	**1.00**
week 5 – week 9	-2.39	0.49	-3.21	to	-1.59	Inf	**1.00**
week 5 – week 10	-1.65	0.49	-2.45	to	-0.83	1124.00	**1.00**
week 5 – week 11	-2.61	0.49	-3.40	to	-1.80	Inf	**1.00**
week 5 – week 12	-2.89	0.49	-3.70	to	-2.06	Inf	**1.00**

week 5 – week 13	-2.74	0.63	-3.78	to	-1.71	17999.00	**1.00**
week 6 – week 7	-0.06	0.48	-0.85	to	0.71	1.21	0.55
week 6 – week 8	0.19	0.46	-0.57	to	0.93	0.50	0.34
week 6 – week 9	-0.66	0.48	-1.44	to	0.12	11.41	0.92
week 6 – week 10	0.09	0.48	-0.70	to	0.87	0.72	0.42
week 6 – week 11	-0.87	0.47	-1.65	to	-0.11	30.97	**0.97**
week 6 – week 12	-1.15	0.48	-1.94	to	-0.37	114.38	**0.99**
week 6 – week 13	-1.00	0.63	-2.06	to	0.01	18.38	0.95
week 7 – week 8	0.25	0.48	-0.54	to	1.03	0.42	0.30
week 7 – week 9	-0.60	0.48	-1.38	to	0.19	8.62	0.90
week 7 – week 10	0.15	0.48	-0.64	to	0.93	0.60	0.38
week 7 – week 11	-0.81	0.48	-1.59	to	-0.02	20.66	**0.95**
week 7 – week 12	-1.09	0.49	-1.89	to	-0.30	65.18	**0.98**
week 7 – week 13	-0.94	0.62	-1.97	to	0.07	15.01	0.94
week 8 – week 9	-0.84	0.47	-1.62	to	-0.08	27.30	**0.96**
week 8 – week 10	-0.10	0.47	-0.87	to	0.68	1.39	0.58
week 8 – week 11	-1.06	0.46	-1.83	to	-0.31	85.96	**0.99**
week 8 – week 12	-1.34	0.48	-2.12	to	-0.56	332.33	**1.00**
week 8 – week 13	-1.19	0.62	-2.23	to	-0.18	37.46	**0.97**
week 9 – week 10	0.75	0.48	-0.03	to	1.52	0.06	0.06
week 9 – week 11	-0.21	0.47	-0.98	to	0.54	2.12	0.68
week 9 – week 12	-0.49	0.48	-1.28	to	0.31	5.44	0.84
week 9 – week 13	-0.34	0.62	-1.38	to	0.67	2.44	0.71
week 10 – week 11	-0.96	0.47	-1.73	to	-0.18	45.75	**0.98**
week 10 – week 12	-1.24	0.48	-2.03	to	-0.45	194.65	**0.99**
week 10 – week 13	-1.09	0.62	-2.13	to	-0.08	25.28	**0.96**
week 11 – week 12	-0.28	0.47	-1.05	to	0.48	2.62	0.72
week 11 – week 13	-0.13	0.62	-1.15	to	0.88	1.38	0.58
week 12 – week 13	0.15	0.61	-0.86	to	1.15	0.67	0.40

*bold = significant, with significant differences when the posterior probability exceeds the 95% CI (CI does not overlap with 0)

Journal of Experimental Biology • Supplementary information

Table S2. Treatment week comparisons of 15°C treatment effect size compared to a constant 10°C for imaging dataset. The difference in effect size between the warm (15°C) and baseline treatment (10°C) was compared between treatment weeks with one-sided hypothesis tests. Estimates in log odds represent the change in the estimated mean difference between treatments (as reported in Table 1) in one week to the next, with the estimated error (Est. Error) and the 95% confidence interval (CI). The evidence ratio (Evid. Ratio) and posterior probability (Post. prob) report the likelihood of a real difference existing (HA) versus no effect (H0). Negative estimates mean a bigger change in the later week compared to the earlier week.

Comparison	Estimate	Est. Error	95% CI			Evid. Ratio	Post. prob*
week 2 – week 3	-0.78	0.62	-1.78	to	0.23	8.81	0.90
week 2 – week 4	0.10	0.60	-0.86	to	1.09	0.76	0.43
week 2 – week 5	-1.69	0.62	-2.70	to	-0.66	210.76	**1.00**
week 2 – week 6	-1.11	0.61	-2.09	to	-0.09	25.01	**0.96**
week 2 – week 7	-1.55	0.60	-2.52	to	-0.55	147.76	**0.99**
week 2 – week 8	-1.67	0.60	-2.65	to	-0.68	294.08	**1.00**
week 2 – week 9	-1.37	0.61	-2.35	to	-0.37	73.69	**0.99**
week 2 – week 10	-1.99	0.62	-2.98	to	-0.98	719.00	**1.00**
week 2 – week 11	-3.24	0.65	-4.30	to	-2.19	Inf	**1.00**
week 2 – week 12	-2.17	0.66	-3.25	to	-1.09	1383.62	**1.00**
week 2 – week 13	-9.76	5.39	-20.29	to	-3.15	17999.00	**1.00**
week 3 – week 4	0.89	0.59	-0.08	to	1.87	0.07	0.07
week 3 – week 5	-0.91	0.61	-1.91	to	0.10	13.79	0.93
week 3 – week 6	-0.32	0.60	-1.29	to	0.67	2.47	0.71
week 3 – week 7	-0.76	0.59	-1.72	to	0.20	9.59	0.91
week 3 – week 8	-0.89	0.59	-1.84	to	0.09	13.75	0.93
week 3 – week 9	-0.58	0.59	-1.55	to	0.39	5.38	0.84
week 3 – week 10	-1.21	0.61	-2.23	to	-0.21	37.14	**0.97**
week 3 – week 11	-2.46	0.64	-3.51	to	-1.40	4499.00	**1.00**
week 3 – week 12	-1.38	0.66	-2.47	to	-0.32	60.22	**0.98**
week 3 – week 13	-8.98	5.40	-19.46	to	-2.33	1124.00	**1.00**
week 4 – week 5	-1.79	0.60	-2.77	to	-0.81	599.00	**1.00**
week 4 – week 6	-1.21	0.56	-2.14	to	-0.28	57.25	**0.98**
week 4 – week 7	-1.65	0.57	-2.59	to	-0.72	438.02	**1.00**
week 4 – week 8	-1.77	0.56	-2.69	to	-0.84	691.31	**1.00**
week 4 – week 9	-1.47	0.57	-2.41	to	-0.53	173.76	**0.99**
week 4 – week 10	-2.10	0.59	-3.06	to	-1.13	1635.36	**1.00**
week 4 – week 11	-3.34	0.62	-4.38	to	-2.33	Inf	**1.00**
week 4 – week 12	-2.27	0.63	-3.33	to	-1.25	5999.00	**1.00**
week 4 – week 13	-9.86	5.39	-20.37	to	-3.23	Inf	**1.00**
week 5 – week 6	0.58	0.60	-0.38	to	1.56	0.19	0.16
week 5 – week 7	0.14	0.59	-0.83	to	1.11	0.67	0.40
week 5 – week 8	0.02	0.60	-0.96	to	1.00	0.95	0.49
week 5 – week 9	0.32	0.60	-0.67	to	1.30	0.41	0.29
week 5 – week 10	-0.31	0.61	-1.29	to	0.68	2.29	0.70
week 5 – week 11	-1.55	0.64	-2.60	to	-0.51	122.29	**0.99**
week 5 – week 12	-0.48	0.66	-1.56	to	0.58	3.28	0.77
week 5 – week 13	-8.07	5.39	-18.58	to	-1.43	165.67	**0.99**
week 6 – week 7	-0.44	0.57	-1.39	to	0.49	3.65	0.78
week 6 – week 8	-0.56	0.55	-1.46	to	0.34	5.68	0.85

week 6 – week 9	-0.26	0.58	-1.22	to	0.68	2.08	0.68
week 6 – week 10	-0.89	0.59	-1.85	to	0.08	14.67	0.94
week 6 – week 11	-2.13	0.62	-3.16	to	-1.12	1799.00	**1.00**
week 6 – week 12	-1.06	0.64	-2.15	to	-0.03	20.58	**0.95**
week 6 – week 13	-8.66	5.40	-19.14	to	-2.03	579.65	**1.00**
week 7 – week 8	-0.12	0.56	-1.03	to	0.79	1.44	0.59
week 7 – week 9	0.18	0.57	-0.76	to	1.11	0.60	0.38
week 7 – week 10	-0.45	0.59	-1.43	to	0.52	3.61	0.78
week 7 – week 11	-1.69	0.62	-2.70	to	-0.68	299.00	**1.00**
week 7 – week 12	-0.62	0.64	-1.66	to	0.41	5.11	0.84
week 7 – week 13	-8.22	5.39	-18.74	to	-1.55	232.77	**1.00**
week 8 – week 9	0.30	0.57	-0.64	to	1.23	0.41	0.29
week 8 – week 10	-0.33	0.58	-1.27	to	0.63	2.58	0.72
week 8 – week 11	-1.57	0.62	-2.59	to	-0.58	168.81	**0.99**
week 8 – week 12	-0.50	0.64	-1.56	to	0.55	3.67	0.79
week 8 – week 13	-8.09	5.40	-18.70	to	-1.42	182.67	**0.99**
week 9 – week 10	-0.63	0.58	-1.57	to	0.32	6.28	0.86
week 9 – week 11	-1.87	0.62	-2.88	to	-0.86	499.00	**1.00**
week 9 – week 12	-0.80	0.64	-1.84	to	0.24	8.71	0.90
week 9 – week 13	-8.39	5.39	-18.90	to	-1.77	390.30	**1.00**
week 10 – week 11	-1.24	0.61	-2.23	to	-0.26	49.14	**0.98**
week 10 – week 12	-0.17	0.63	-1.23	to	0.85	1.55	0.61
week 10 – week 13	-7.77	5.40	-18.26	to	-1.12	99.00	**0.99**
week 11 – week 12	1.07	0.64	0.02	to	2.11	0.05	0.05
week 11 – week 13	-6.52	5.40	-17.06	to	0.13	16.65	0.94
week 12 – week 13	-7.60	5.40	-18.12	to	-0.94	66.42	**0.99**

*bold = significant, with significant differences when the posterior probability exceeds the 95% CI (CI does not overlap with 0)

Table S3. Treatment week comparisons of 5°C treatment effect size compared to a constant 10°C for hatching dataset. The difference in effect size between the cold (5°C) and baseline treatment (10°C) was compared between treatment weeks with one-sided hypothesis tests. Estimates in April days represent the change in the estimated mean difference between treatments (as reported in Table 2) in one week to the next, with the estimated error (Est. Error) and the 95% confidence interval (CI). The evidence ratio (Evid. Ratio) and posterior probability (Post. prob) report the likelihood of a real difference existing (HA) versus no effect (H0). Negative estimates mean a bigger change in the later week compared to the earlier week.

Comparison	Estimate	Est. Error	95% CI			Evid. Ratio	Post. prob*
week 2 – week 3	-1.40	1.35	-3.64	to	0.79	5.76	0.85
week 2 – week 4	-0.45	1.35	-2.64	to	1.75	1.74	0.63
week 2 – week 5	-1.04	1.25	-3.12	to	1.01	4.03	0.80
week 2 – week 6	-3.64	1.32	-5.81	to	-1.49	249.00	**1.00**
week 2 – week 7	-0.58	1.24	-2.60	to	1.44	2.16	0.68
week 2 – week 8	-4.73	1.33	-6.91	to	-2.55	2249.00	**1.00**
week 2 – week 9	-5.24	1.27	-7.32	to	-3.13	Inf	**1.00**
week 2 – week 10	-6.48	1.33	-8.66	to	-4.31	Inf	**1.00**
week 2 – week 11	-6.05	1.35	-8.28	to	-3.86	Inf	**1.00**
week 2 – week 12	-8.22	1.26	-10.31	to	-6.16	Inf	**1.00**
week 2 – week 13	-8.77	1.62	-11.44	to	-6.11	Inf	**1.00**
week 3 – week 4	0.95	1.45	-1.42	to	3.29	0.35	0.26
week 3 – week 5	0.35	1.39	-1.93	to	2.63	0.65	0.39
week 3 – week 6	-2.25	1.43	-4.60	to	0.11	16.18	0.94
week 3 – week 7	0.81	1.36	-1.44	to	3.06	0.38	0.27
week 3 – week 8	-3.33	1.44	-5.68	to	-0.98	103.65	**0.99**
week 3 – week 9	-3.85	1.39	-6.14	to	-1.55	299.00	**1.00**
week 3 – week 10	-5.09	1.43	-7.47	to	-2.78	8999.00	**1.00**
week 3 – week 11	-4.65	1.46	-7.06	to	-2.28	1284.71	**1.00**
week 3 – week 12	-6.82	1.38	-9.13	to	-4.56	Inf	**1.00**
week 3 – week 13	-7.38	1.71	-10.16	to	-4.59	Inf	**1.00**
week 4 – week 5	-0.59	1.34	-2.78	to	1.58	2.07	0.67
week 4 – week 6	-3.19	1.41	-5.53	to	-0.87	88.11	**0.99**
week 4 – week 7	-0.13	1.36	-2.39	to	2.10	1.14	0.53
week 4 – week 8	-4.28	1.44	-6.63	to	-1.92	561.50	**1.00**
week 4 – week 9	-4.79	1.37	-7.01	to	-2.56	1799.00	**1.00**
week 4 – week 10	-6.03	1.43	-8.38	to	-3.66	Inf	**1.00**
week 4 – week 11	-5.60	1.44	-7.95	to	-3.25	8999.00	**1.00**
week 4 – week 12	-7.77	1.35	-9.97	to	-5.56	Inf	**1.00**
week 4 – week 13	-8.32	1.70	-11.08	to	-5.57	Inf	**1.00**
week 5 – week 6	-2.60	1.32	-4.80	to	-0.42	38.47	**0.97**
week 5 – week 7	0.46	1.27	-1.62	to	2.55	0.55	0.35
week 5 – week 8	-3.68	1.36	-5.92	to	-1.45	332.33	**1.00**
week 5 – week 9	-4.20	1.28	-6.29	to	-2.09	1124.00	**1.00**
week 5 – week 10	-5.44	1.33	-7.66	to	-3.24	Inf	**1.00**
week 5 – week 11	-5.01	1.35	-7.24	to	-2.81	8999.00	**1.00**
week 5 – week 12	-7.17	1.29	-9.27	to	-5.07	Inf	**1.00**
week 5 – week 13	-7.73	1.65	-10.45	to	-5.05	Inf	**1.00**
week 6 – week 7	3.06	1.32	0.88	to	5.23	0.01	0.01
week 6 – week 8	-1.08	1.40	-3.42	to	1.17	3.56	0.78

Journal of Experimental Biology • Supplementary information

week 6 – week 9	-1.60	1.35	-3.84	to	0.63	7.67	0.88
week 6 – week 10	-2.84	1.38	-5.11	to	-0.60	51.94	**0.98**
week 6 – week 11	-2.41	1.40	-4.72	to	-0.10	22.38	**0.96**
week 6 – week 12	-4.57	1.33	-6.77	to	-2.41	8999.00	**1.00**
week 6 – week 13	-5.13	1.67	-7.86	to	-2.39	749.00	**1.00**
week 7 – week 8	-4.14	1.33	-6.30	to	-1.93	599.00	**1.00**
week 7 – week 9	-4.66	1.28	-6.73	to	-2.57	8999.00	**1.00**
week 7 – week 10	-5.90	1.31	-8.04	to	-3.75	8999.00	**1.00**
week 7 – week 11	-5.47	1.33	-7.66	to	-3.31	Inf	**1.00**
week 7 – week 12	-7.64	1.26	-9.73	to	-5.57	Inf	**1.00**
week 7 – week 13	-8.19	1.63	-10.82	to	-5.47	Inf	**1.00**
week 8 – week 9	-0.51	1.38	-2.77	to	1.75	1.85	0.65
week 8 – week 10	-1.76	1.43	-4.08	to	0.59	8.44	0.89
week 8 – week 11	-1.32	1.41	-3.66	to	0.99	4.83	0.83
week 8 – week 12	-3.49	1.37	-5.72	to	-1.21	172.08	**0.99**
week 8 – week 13	-4.04	1.68	-6.80	to	-1.27	124.00	**0.99**
week 9 – week 10	-1.24	1.36	-3.45	to	0.98	4.73	0.83
week 9 – week 11	-0.81	1.39	-3.14	to	1.47	2.60	0.72
week 9 – week 12	-2.98	1.28	-5.08	to	-0.87	112.92	**0.99**
week 9 – week 13	-3.53	1.63	-6.19	to	-0.89	66.67	**0.99**
week 10 – week 11	0.43	1.39	-1.83	to	2.72	0.60	0.38
week 10 – week 12	-1.73	1.35	-3.95	to	0.49	9.42	0.90
week 10 – week 13	-2.29	1.69	-5.09	to	0.46	10.97	0.92
week 11 – week 12	-2.17	1.36	-4.43	to	0.08	16.51	0.94
week 11 – week 13	-2.72	1.69	-5.49	to	0.06	17.60	0.95
week 12 – week 13	-0.55	1.64	-3.23	to	2.13	1.71	0.63

*bold = significant, with significant differences when the posterior probability exceeds the 95% CI (CI does not overlap with 0)

Table S4. Treatment week comparisons of 15°C treatment effect size compared to a constant 10°C for hatching dataset. The difference in effect size between the warm (15°C) and baseline treatment (10°C) was compared between treatment weeks with one-sided hypothesis tests. Estimates in April days represent the change in the estimated mean difference between treatments (as reported in Table 2) in one week to the next, with the estimated error (Est. Error) and the 95% confidence interval (CI). The evidence ratio (Evid. Ratio) and posterior probability (Post. prob) report the likelihood of a real difference existing (HA) versus no effect (H0). Negative estimates mean a bigger change in the later week compared to the earlier week.

Comparison	Estimate	Est. Error	95% CI			Evid. Ratio	Post. Prob*
week 2 – week 3	2.10	1.56	-0.49	to	4.65	0.09	0.09
week 2 – week 4	0.78	1.52	-1.74	to	3.28	0.44	0.31
week 2 – week 5	-1.72	1.46	-4.12	to	0.68	7.67	0.88
week 2 – week 6	-1.58	1.50	-4.08	to	0.89	5.91	0.86
week 2 – week 7	-5.42	1.47	-7.80	to	-3.03	4499.00	**1.00**
week 2 – week 8	-4.09	1.56	-6.61	to	-1.52	208.30	**1.00**
week 2 – week 9	-2.70	1.50	-5.14	to	-0.28	28.32	**0.97**
week 2 – week 10	-2.90	1.52	-5.36	to	-0.43	36.82	**0.97**
week 2 – week 11	-3.04	1.52	-5.56	to	-0.55	39.72	**0.98**
week 2 – week 12	-2.31	1.48	-4.78	to	0.11	16.34	0.94
week 2 – week 13	1.54	1.86	-1.54	to	4.54	0.25	0.20
week 3 – week 4	-1.32	1.68	-4.03	to	1.47	3.77	0.79
week 3 – week 5	-3.81	1.63	-6.50	to	-1.12	74.00	**0.99**
week 3 – week 6	-3.67	1.67	-6.39	to	-0.94	63.75	**0.98**
week 3 – week 7	-7.52	1.63	-10.16	to	-4.83	Inf	**1.00**
week 3 – week 8	-6.19	1.67	-8.89	to	-3.43	2249.00	**1.00**
week 3 – week 9	-4.80	1.66	-7.50	to	-2.05	390.30	**1.00**
week 3 – week 10	-5.00	1.66	-7.70	to	-2.29	345.15	**1.00**
week 3 – week 11	-5.13	1.66	-7.82	to	-2.42	449.00	**1.00**
week 3 – week 12	-4.41	1.62	-7.07	to	-1.78	242.24	**1.00**
week 3 – week 13	-0.55	1.97	-3.74	to	2.67	1.60	0.62
week 4 – week 5	-2.49	1.57	-5.06	to	0.07	17.00	0.94
week 4 – week 6	-2.36	1.62	-5.07	to	0.28	13.24	0.93
week 4 – week 7	-6.20	1.59	-8.79	to	-3.57	2999.00	**1.00**
week 4 – week 8	-4.87	1.67	-7.62	to	-2.16	359.00	**1.00**
week 4 – week 9	-3.48	1.63	-6.18	to	-0.84	61.94	**0.98**
week 4 – week 10	-3.68	1.64	-6.37	to	-1.01	68.77	**0.99**
week 4 – week 11	-3.81	1.63	-6.50	to	-1.11	90.84	**0.99**
week 4 – week 12	-3.09	1.62	-5.78	to	-0.51	35.44	**0.97**
week 4 – week 13	0.77	1.95	-2.44	to	3.97	0.52	0.34
week 5 – week 6	0.14	1.56	-2.42	to	2.68	0.87	0.47
week 5 – week 7	-3.70	1.51	-6.21	to	-1.24	125.76	**0.99**
week 5 – week 8	-2.37	1.62	-5.01	to	0.27	13.38	0.93
week 5 – week 9	-0.98	1.55	-3.52	to	1.52	2.91	0.74
week 5 – week 10	-1.18	1.55	-3.72	to	1.39	3.68	0.79
week 5 – week 11	-1.32	1.57	-3.89	to	1.26	4.04	0.80
week 5 – week 12	-0.60	1.54	-3.12	to	1.92	1.88	0.65
week 5 – week 13	3.26	1.90	0.14	to	6.36	0.04	0.04
week 6 – week 7	-3.84	1.57	-6.38	to	-1.26	127.57	**0.99**
week 6 – week 8	-2.51	1.61	-5.12	to	0.14	16.18	0.94

week 6 – week 9	-1.12	1.59	-3.75	to	1.47	3.24	0.76
week 6 – week 10	-1.32	1.62	-4.01	to	1.32	3.95	0.80
week 6 – week 11	-1.46	1.61	-4.06	to	1.24	4.53	0.82
week 6 – week 12	-0.74	1.58	-3.37	to	1.84	2.19	0.69
week 6 – week 13	3.12	1.94	-0.05	to	6.31	0.06	0.05
week 7 – week 8	1.33	1.59	-1.25	to	3.96	0.25	0.20
week 7 – week 9	2.72	1.55	0.15	to	5.26	0.04	0.04
week 7 – week 10	2.52	1.56	-0.05	to	5.07	0.06	0.05
week 7 – week 11	2.38	1.56	-0.14	to	4.97	0.07	0.06
week 7 – week 12	3.11	1.54	0.62	to	5.63	0.02	0.02
week 7 – week 13	6.97	1.89	3.89	to	10.06	0.00	0.00
week 8 – week 9	1.39	1.65	-1.34	to	4.07	0.24	0.19
week 8 – week 10	1.19	1.65	-1.54	to	3.88	0.30	0.23
week 8 – week 11	1.06	1.64	-1.63	to	3.78	0.35	0.26
week 8 – week 12	1.78	1.65	-0.93	to	4.44	0.16	0.14
week 8 – week 13	5.64	1.95	2.45	to	8.83	0.00	0.00
week 9 – week 10	-0.20	1.58	-2.80	to	2.39	1.24	0.55
week 9 – week 11	-0.34	1.61	-2.96	to	2.30	1.43	0.59
week 9 – week 12	0.39	1.57	-2.18	to	2.96	0.68	0.40
week 9 – week 13	4.25	1.94	1.05	to	7.41	0.01	0.01
week 10 – week 11	-0.14	1.60	-2.72	to	2.54	1.17	0.54
week 10 – week 12	0.59	1.59	-1.99	to	3.20	0.54	0.35
week 10 – week 13	4.44	1.94	1.30	to	7.59	0.01	0.01
week 11 – week 12	0.72	1.59	-1.93	to	3.32	0.47	0.32
week 11 – week 13	4.58	1.95	1.39	to	7.77	0.01	0.01
week 12 – week 13	3.86	1.91	0.76	to	6.99	0.02	0.02

*bold = significant, with significant differences when the posterior probability exceeds the 95% CI (CI does not overlap with 0)
